# Healthcare and community stakeholders’ perceptions of barriers and facilitators to implementing a behavioral activation intervention for people with dementia and depression: a qualitative study using Normalization Process Theory

**DOI:** 10.1186/s12877-023-04522-9

**Published:** 2023-12-07

**Authors:** Frida Svedin, Oscar Blomberg, Anders Brantnell, Paul Farrand, Anna Cristina Åberg, Joanne Woodford

**Affiliations:** 1 Healthcare Sciences and E-Health, Department of Women’s and Children’s Health, Uppsala University, Uppsala, 751 85 Sweden; 2 Industrial Engineering and Management, Department of Civil and Industrial Engineering, Uppsala University, 751 21 Uppsala, Sweden; 3 Clinical Education, Development and Research (CEDAR); Psychology, University of Exeter, Perry Road, Devon, EX4 4QG UK; 4 Clinical Geriatrics, Department of Public Health and Caring Sciences, Uppsala University, 751 85 Uppsala, Sweden; 5 Medical Sciences, School of Health and Welfare, Dalarna University, 791 88 Falun, Sweden

**Keywords:** Dementia, Depression, Mental Health, Behavioral Activation, Normalization Process Theory, Intervention Development

## Abstract

**Background:**

Depression is commonly experienced by people with dementia, and associated with lower quality of life and functional decline. However, access to evidence-based psychological interventions for people with dementia and depression is limited. One potential solution is guided low-intensity behavioral activation. Following the new Medical Research Council Framework, considering factors such as potential barriers and facilitators to implementation is recommended during the development of new interventions. Aims of this study were to: (1) develop an understanding of existing healthcare and community support in the Swedish context for people with dementia and their informal caregivers; and (2) identify barriers and facilitators to intervention uptake informed by Normalization Process Theory.

**Methods:**

Semi-structured interviews and focus groups were held with healthcare (*n* = 18) and community (*n* = 7) stakeholders working with people with dementia and/or informal caregivers. Interview questions were informed by Normalization Process Theory. Data was analysed utilizing a two-step deductive analysis approach using the Normalization Process Theory coding manual, with inductive categories applied to data related to the main mechanisms of the theory, but not captured by its sub-constructs.

**Results:**

Ten deductive and three inductive categories related to three Normalization Process Theory primary mechanisms (*Coherence, Cognitive Participation, and Collective Action*) were identified. Identified barriers to intervention uptake included: (1) additional burden for informal caregivers; (2) lack of appropriate workforce to provide guidance; (3) lack of time and financial resources; (4) people with dementia not recognising their diagnosis of dementia and/or a need for support; and (5) stigma. Identified facilitators to intervention uptake included: (1) intervention has potential to fill a large psychological treatment gap in Sweden; (2) objectives and potential benefits understood and agreed by most stakeholders; and (3) some healthcare professionals recognized their potential role in providing intervention guidance.

**Conclusions:**

Several barriers and facilitators for future implementation, specific to the intervention, individuals and families, as well as professionals, were identified during intervention development. Barriers were mapped into evidence-based implementation strategies, which will be adopted to overcome identified barriers. A feasibility study further examining implementation potential, acceptability and feasibility, alongside clinical, methodological, and procedural uncertainties associated with the intervention will be conducted.

**Trial registration:**

Not applicable.

**Supplementary Information:**

The online version contains supplementary material available at 10.1186/s12877-023-04522-9.

## Background

The number of people living with dementia (PWD) continues to rise worldwide [1]. By 2060, dementia will be the condition with the highest proportional increase in serious health related suffering globally [2]. Dementia represents one of the greatest global health and social care challenges of the 21^st^ Century [3], negatively impacting individuals and informal caregivers (caregivers) [4]. Further, dementia places significant burden on healthcare systems and wider society globally [1], with the estimated cost of dementia predicted to rise from $275 billion in 2019 to between $1.6 and 2.4 trillion by 2050 [5].

Mental health difficulties are commonly experienced by PWD, with up to 41% experiencing depression [6, 7]. Depression in PWD is associated with poor quality of life, reduction in daily activities, and functional decline [8]. Evidence-based psychological interventions (e.g., cognitive behavioral therapy (CBT)) exist for PWD and depression [7], however, access is limited [9, 10]. Overall, healthcare utilization among PWD and caregivers is low and associated with barriers including geographical distance, stigma [11], lack of knowledge of available services, lack of tailored services, limited healthcare professional (HCP) time, and poor financial resources [12].

One solution to improve access to psychological interventions is low-intensity CBT (LI-CBT) [13, 14]. LI-CBT represents a single evidence-based CBT technique being adopted to target a specific mental health problem [15]. Techniques are delivered in self-help format via health technology such as internet administered, audiobooks, or written materials [13]. Evidence suggests providing trained HCP guidance to people using LI-CBT is associated with increased effectiveness [16, 17]. Given LI-CBT techniques are delivered via health technology, it represents a flexible way to deliver psychological interventions [13] and may overcome barriers including geographical distance, stigma [11], and limited HCP time [13].

Simple behavioral activation (BA) is an example of an evidence-based LI-CBT technique for depression. Simple BA adopts a structured and graded approach to increase engagement in pleasurable, routine, and necessary activities to target behavioral avoidance [18]. Behavioral avoidance has been identified as a mechanism that can lead to depression [19] which is exacerbated by dementia symptoms [20]. Given PWD articulate living well with dementia as being able to find enjoyment in life, participate in usual activities, and retain social connectiveness [21], supporting re-engagement in activities is of particular importance to enhance psychological wellbeing.

Given the promise of a guided low-intensity behavioral activation (LI-BA) intervention for PWD and depression, a research program informed by Phase I of the Medical Research Council (MRC) framework [22] for complex interventions has been undertaken in the United Kingdom (UK) [23, 24]. However, before testing the intervention in Sweden, adaptation is needed to improve the intervention-context fit, whilst maintaining consistency with the interventions’ evidence-based components [25]. In accordance with the MRC framework [22], to increase the likelihood of future intervention implementation in real-world settings, it is essential to consider implementation potential (i.e., the likelihood of intervention uptake and implementation in practice) throughout intervention development [26, 27]. Considering core components of the MRC framework [22] such as context and stakeholder involvement, may facilitate our understanding of factors related to future implementation. For example, to identify potential barriers and facilitators for intervention uptake [28], overcome implementation barriers [22], and enhance intervention acceptability and feasibility [29].

To identify potential implementation barriers and facilitators during intervention development the use of an implementation theory, such as Normalization Process Theory (NPT), may be helpful [30]. NPT is a middle-range sociological implementation theory, focusing on social action, representing the individual and collective work stakeholders have to do to successfully implement complex interventions into real-world setting [31, 32]. NPT may be used to identify barriers and facilitators for normalizing and incorporating complex interventions to ensure they can be embedded into routine practice. NPT includes four primary NPT mechanisms: (1) *Coherence* (sense-making); (2) *Cognitive Participation* (commitment and engagement); (3) *Collective Action* (work to enact); and (4) *Reflexive Monitoring* (appraisal) (Table 1) [32]. By understanding implementation barriers and facilitators and the work stakeholders have to do to normalize complex interventions into routine practice, NPT provides a framework for intervention development by identifying potential relevant contextual issues and improving complex interventions through increasing implementation potential [27, 33, 34].

**Table 1:** Overview of primary Normalization Process Theory (NPT) mechanisms and sub-constructs

Primary NPT mechanism	Sub-construct
*Coherence*	Differentiation
	Communal specification
	Individual specification
	Internalization
*Cognitive Participation*	Initiation
	Enrolment
	Legitimation
	Activation
*Collective Action*	Interactional workability
	Relational integration
	Skill-set workability
	Contextual integration
*Reflexive Monitoring*	Systematization
	Communal appraisal
	Individual appraisal
	Reconfiguration

## Aim

This study is part of a wider research project [35] with the overall aim of adapting a guided LI-BA intervention for PWD and depression and caregivers, and enhance future implementation potential for the Swedish context.

Specific objectives were to: (1) develop an understanding of the existing healthcare and community support in the Swedish context for PWD and caregivers; and (2) identify barriers and facilitators to intervention uptake informed by NPT.

## Methods

### Qualitative approach and research paradigm

Pragmatism was adopted as the qualitative approach and research paradigm [36, 37],

meaning (1) study design decisions were based on “what will work best” in answering the study objectives; (2) study aims were based on finding a solution to a “real-world problem”; and (3) findings may be considered applicable or transferable to other similar contexts [36, 37].

### Researcher characteristics and reflexivity

Analysis was primarily conducted by FS and OB and supervised by ACÅ. FS is a female doctoral candidate (BSc Biomedicine, MSc Public Health). OB is a male doctoral candidate (BSc Sport Science, MSc Public Health). FS and OB were trained in content analysis by ACÅ and attended postgraduate qualitative training. ACÅ is a female professor in Medical Science, with a focus on Geriatrics and Implementation Science. ACÅ led the development of the Swedish version of the NPT outcome measurement [38], has extensive experience of qualitative research, and led data analysis workshops and training sessions. JW is a female researcher with a PhD in Psychology and extensive experience in conducting qualitative research. JW is the principal investigator of the study and led peer examination discussions. PF and AB provided peer examination. PF is a male professor in Evidence-Based Psychological Practice, has extensive experience of qualitative research, and is an expert in LI-CBT. AB is a male researcher with a PhD in Medical Science, with a focus on healthcare innovation implementation. All have been part of the research team since study setup.

### Study design

A qualitative study design informed by principles from co-design [39] and participatory action research [40], placing key stakeholders at the centre of the research process [39]. Principles of co-design were informed by definitions that consider co-design to be a component of co-creation, whereby designers (i.e., the research team) work in collaboration with people not trained in design (i.e., members of the public, healthcare workers, non-profit organization workers) [41] to design some form of intervention or service or solution. Methods and results are reported in accordance with the Standards for Reporting Qualitative Research [42] (Additional file 1).

### Context

There are approximately 160 000 PWD in Sweden [43]. Sweden is divided into 21 regions and 290 municipalities, with regions primarily responsible for general healthcare, for example primary care and memory clinics [44], and municipalities responsible for general and specialized care such as home care services, day care, and caregiver support [45].

### Study participants

Eligible HCPs and community stakeholders were: (1) ≥ 18 years old; (2) working with PWD and/or caregivers; (3) able to understand, read and write in Swedish and/or English; and (4) living in Sweden.

### Recruitment

HCPs and community stakeholders were recruited using purposeful variation [46], convenience [47], and snowball sampling [48], via in-person networks, and advertising. HCPs were recruited from five regions in East Middle Sweden [Uppsala, Stockholm, Västmanland, Södermanland, and Örebro] and community stakeholders were recruited across Sweden. Study invitation packs were sent via post or e-mail to potential participants, including: (1) study invitation letter; (2) study information sheet; (3) reply slip; (4) reasons for non-participation questionnaire; and (5) stamped addressed envelope.

### Informed consent and eligibility screening

Written informed consent was obtained and an eligibility screen was conducted. Eligible participants completed a background questionnaire including age, gender, profession, length of time in that profession, professional qualifications, and length of time working with PWD and/or caregivers.

### Reasons for non-participation

Those declining participation were asked to complete an anonymous reason for non-participation form consisting of a closed, multiple-choice question regarding reasons for non-participation [23, 49].

### Intervention

The clinical protocol and intervention delivery model for the LI-BA intervention developed in the UK is published elsewhere [24, 35]. The intervention is developed for community-dwelling people with mild-to-moderate dementia experiencing depression and is based on a simple BA approach [50]. PWD are supported by a caregiver to gradually re-engage in activities they used to do, but have stopped doing, and/or identify new activities of similar value, importance, or meaning. The PWD and caregiver receives guidance (face-to-face and telephone) from an intervention guide (e.g., trained HCP). BA techniques are delivered via two written workbooks – one for PWD and one for caregivers. Supervisors provide training and weekly supervision to intervention guides.

### Data collection

One HCP declined participation, stating lack of time as reason. Informed consent was provided by 19 HCPs, one withdrew participation due to re-allocation of resources during the COVID-19 pandemic. The remaining 18 HCPs and seven community stakeholders participated in focus groups and semi-structured interviews (May 2021 to October 2021). Prior to focus groups and interviews, stakeholders were provided with a written summary of the intervention delivery model developed in the UK and translated workbooks in Swedish. Three focus groups (60–91 min) with three, four, and five HCPs (*n* = 12) respectively were conducted. Semi-structured interviews (43–97 min) were conducted with HCPs unable to attend focus groups (*n* = 6) and all community stakeholders (*n* = 7). Focus groups and interviews were conducted by two research team members (FS, OB) following an interview guide informed by the three primary NPT mechanisms: (1) *Coherence*; (2) *Cognitive Participation*; and (3) *Collective Action* (Additional file 2) [31, 51]. We did not include the fourth primary NPT mechanism (*Reflexive Monitoring*) given this mechanism refers to how individuals and groups appraise how the intervention affects them in practice [52], which may be difficult for stakeholders to consider during early intervention development [34]. Examples of interview questions and corresponding primary NPT mechanisms are provided in Table 2.

**Table 2:** Example of interview questions and corresponding primary Normalization Process Theory (NPT) mechanism

Primary NPT Mechanism	Interview Question
Coherence	• How does the support provided in the intervention differ from the support people with dementia are currently receiving? • How would you describe the purpose of the intervention? • What impact do you think the intervention can have on people with dementia and informal caregivers?
Cognitive Participation	• What type of support do you think people with dementia and informal caregivers need to understand what the intervention is and how it should be used? • What type of support do you think people in your organization need to understand what the intervention is and how it should be used? • What difficulties might people with dementia and informal caregivers experience when using the intervention?
Collective Action	• How would the intervention affect the way of working for the workforce providing the support to people with dementia and informal caregivers? • What can be done to ensure that the intervention becomes part of the everyday work for the workforce providing the support to people with dementia and informal caregivers? • What resources would be needed to ensure that the intervention becomes a part of the everyday work for the workforce providing the support to people with dementia and informal caregivers?

### Participant characteristics

HCPs (*n* = 18) involved in dementia assessment, diagnosis, treatment and/or support participated, with the following professions represented: assistant nurses, dementia care consultants, caregiver consultants, nurses, occupational therapists, physiotherapists, physicians, psychologists, speech therapists, and social workers. The mean age of HCPs was 47 years, the majority were female (94%), with an average of 18 years of experience working with dementia (Table 3). The following regions were represented: Uppsala, Stockholm, Västmanland, Södermanland, and Örebro.

**Table 3:** Sample characteristics of healthcare professionals (*n* = 18)

**Participant**	**Age** (5-year interval)	**Gender** (Male/Female)	**Experience working with dementia** (years)
Healthcare professional 1	60–64	Female	45
Healthcare professional 2	55–59	Female	30
Healthcare professional 3	45–49	Female	17
Healthcare professional 4	40–44	Female	0.5
Healthcare professional 5	40–44	Female	8
Healthcare professional 6	60–64	Female	26
Healthcare professional 7	25–29	Female	2
Healthcare professional 8	60–64	Female	31
Healthcare professional 9	60–64	Female	40
Healthcare professional 10	40–44	Female	8
Healthcare professional 11	40–44	Female	16
Healthcare professional 12	55–59	Female	9
Healthcare professional 13	45–49	Female	12
Healthcare professional 14	55–59	Female	40
Healthcare professional 15	40–44	Female	16
Healthcare professional 16	35–39	Male	1
Healthcare professional 17	25–29	Female	1
Healthcare professional 18	40–44	Female	16

Relevant community stakeholders (*n* = 7) participated and had a mean age of 70 years, the majority were females (71%), with an average of 20 years’ experience working with dementia. Two community stakeholders were employed in a national organisation, the remaining five worked as volunteers on a regional level (Table 4). The following regions were represented: Norrbotten, Uppsala, Stockholm, and Skåne.

**Table 4:** Sample characteristics community stakeholders (*n* = 7)

**Participant**	**Age** (5-year interval)	**Gender** (Male/Female)	**Size of community organisation** (National/Regional)	**Employed or volunteer in community organisation** (Employed/Volunteer)	**Experience working with dementia** (years)
Community stakeholder 1	60–64	Female	National	Employed	10
Community stakeholder 2	70–74	Female	Region	Volunteer	40
Community stakeholder 3	70–74	Female	National	Employed	17
Community stakeholder 4	70–74	Female	Regional	Volunteer	43
Community stakeholder 5	75–79	Male	Regional	Volunteer	8
Community stakeholder 6	75–79	Male	Regional	Volunteer	10
Community stakeholder 7	65–69	Female	Regional	Volunteer	10

### Data processing

Focus groups and interview recordings were transcribed verbatim by an external professional transcriber (*n* = 9) or FS and OB (*n* = 7) and uploaded into NVivo 14 to support data analysis (e.g., organize data and facilitate coding process) [53].

### Data analysis

Step one: a deductive coding approach informed by the NPT coding manual [52] was carried out by FS and OB independently, coding data relevant to the three primary NPT mechanisms: (1) *Coherence*; (2) *Cognitive Participation*; and (3) *Collective Action* [52]. Step two: FS organized codes within each primary NPT mechanism into relevant NPT sub-constructs (categories). Codes relating to primary NPT mechanisms, but unrelated NPT sub-constructs were organized into inductive sub-categories given inductive categories made more sense for the specific implementation topic, i.e., initial intervention development [54]. Within *Coherence*, two sub-constructs (*Communal Specification* and *Individual Specification*) were combined into one construct (*Specification*). Given this study explores future intervention implementation and a specific implementation setting has not yet been identified and stakeholders came from a variety of different settings, differentiating between data referring to individual versus communal specification was difficult. Communal specification refers to a collective agreement between a group of people about the purpose of an intervention and its components, whereas individual specification refers to how people individually understand an intervention and its components [52]. Consequently, when stakeholders discussed the purpose and understanding of the intervention and its components, it was sometimes difficult to know whether they were talking about a group (e.g., co-workers, organization) or themselves. Coding workshops (*n* = 18) were held with FS, OB, and supervised by ACÅ for sense-making and interpretation of the codes and NPT coding manual (e.g., differentiation between codes and which primary NPT mechanism data belongs to, discussing disagreements, and synchronization of codes). Categories and category descriptions were provided in English to ACÅ and JW for peer examination. Categorization workshops were held with FS and ACÅ (*n* = 3), and FS, ACÅ, and JW (*n* = 2).

### Trustworthiness

Trustworthiness was established using: peer examination of supervising researchers (ACÅ and JW) with wider team discussions (FS, OB, JW, PF, AB, ACÅ); independent coding by two researchers (FS and OB); and record keeping [55]. Disconfirming cases and divergent discourses were discussed with supervising researchers (ACÅ and JW). A trustworthiness checklist developed for content analysis was followed [56].

### Public contribution in research

A Public Advisory Group (PAG) consisting of caregivers (*n* = 4) with lived experience of caring for PWD were recruited as research partners. PAG members were aged 44–71 years old, wives and daughters of PWD, with 5–9 years of experience caring for a PWD. PAG members contributed at the ‘Involve’ level, directly working alongside the research team to ensure concerns of the public are listened to during the intervention development [57]. PAG members were involved in sense-making and interpretation of findings for the wider project and helped to co-design the workbooks on the basis of project findings. The impact of public contribution activities will be reported elsewhere.

## Results

Findings are presented in accordance with three (of four) primary NPT mechanisms and applicable sub-constructs (categories): *Coherence* (*Differentiation, Specification,* and *Internalization*); *Cognitive Participation* (*Initiation, Enrolment,* and *Legitimation*), and *Collective Action* (*Interactional Workability, Relational Integration, Skill-set Workability,* and *Contextual Integration*). Analysis also resulted in three inductive categories related to the primary NPT mechanisms: *Relational Interaction* (*Cognitive Participation)*, *Definition and Evaluation of the Situation* (*Cognitive Participation)*, and *Prerequisites for Intervention Interaction* (*Collective Action).* See Table 5 for definitions of primary NPT mechanism and sub-constructs (categories).

**Table 5:** Definitions of primary Normalisation Process Theory (NPT) mechanisms and sub-constructs (categories)

Primary NPT mechanisms and sub-constructs (categories)	Definition
*Coherence* (sense-making)	Individual and collective work to make sense of implementing and integrating the intervention into routine practice
Differentiation	Ability to distinguish the intervention from current practice
Specification	Agreement concerning the purpose of the intervention
Internalization	Understanding the value, benefits, and importance of the intervention
*Cognitive Participation* (commitment and engagement)	Relational work, driven by commitment, to build and sustain the intervention in practice
Initiation	Working to drive the intervention forward
Enrolment	Organizing in order to contribute to the intervention work and to sustain engagement in the intervention
Legitimation	Agreement that the intervention is the right thing to do and that it should be part of routine practice
Relational interaction (*inductive*)	Establishing and maintaining care relationships required for commitment and sustained use of the intervention
Definition and evaluation of the situation (*inductive*)	Understanding, defining, and evaluating of the dyad’s situation and need for the intervention
*Collective Action* (work to enact)	Work to enact the intervention in everyday practice
Interactional workability	Working with each other, and the intervention, to seek to operationalize it in routine practice
Relational integration	Building accountability and maintaining confidence in each other in continued use of the intervention
Skill-set workability	Allocating work around a set of practices (e.g., training) to operationalize the intervention in routine practice
Contextual integration	Allocating resources, protocols, and policies, and procedures to operatize the intervention in routine practice
Prerequisites for intervention interaction (*inductive*)	Prerequisites for the dyad to manage and undertake the intervention in their everyday life

**Fig. 1:**
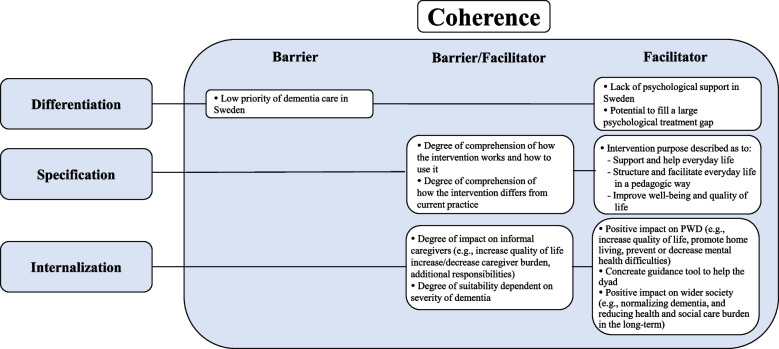
Facilitators and barriers mapped to the sub-constructs of the NPT main mechanism Coherence. PWD = People With Dementia

**Fig. 2:**
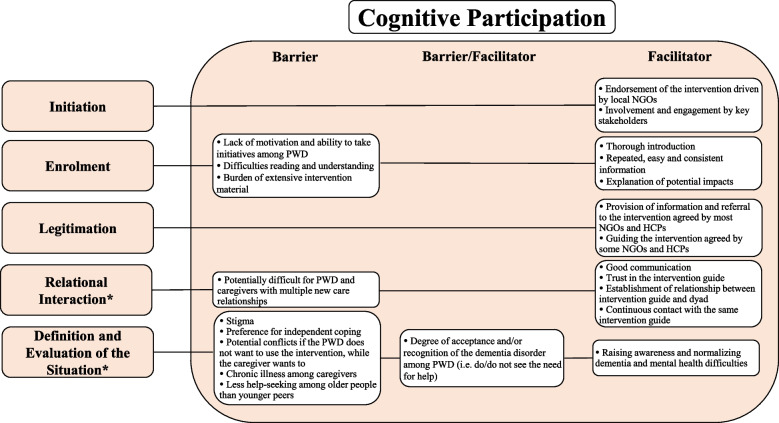
Facilitators and barriers mapped to the sub-constructs of the NPT main mechanism Cognitive Participation. * = Inductive category; HCP = Healthcare Professional; NGO = Non-Governmental Organization; PWD = People With Dementia

**Fig. 3:**
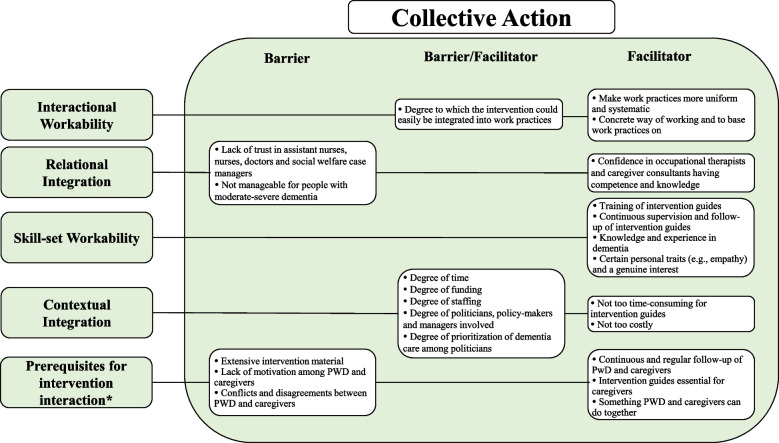
Facilitators and barriers mapped to the sub-constructs of the NPT main mechanism Collective Action. * = Inductive category; HCP = Healthcare Professional; NGO = Non-Governmental Organization; PWD = People With Dementia

Barriers and facilitators were mapped to primary NPT mechanisms and respective sub-constructs (categories) to inform the future implementation processes (see Figs. 1, 2, and 3). As a qualitative study, we did not compare and contrast HCPs and community stakeholders’ concerns. However, type of professional background is provided alongside supporting quotations to aid interpretation.

### Coherence

Data supporting the NPT sub-constructs *Differentiation*, *Specification* (*Individual* and *Communal*), and *Internalization* were identified (Fig. 1).

#### Differentiation

Generally, HCPs and community stakeholders differentiated the intervention from current practice, expressing little to no psychological support is available for PWD and caregivers in Sweden:

*“There is very little support available. That is the problem. There is nothing.”* (Community stakeholder 4)

However, there were two disconfirming cases, with stakeholders considering the intervention to be partially or fully the same as current working practices:

*“I recognize a lot; it is not new.”* (Community stakeholder 2)

Regional variation in support availability was also recognized, with more support being available in larger cities and services limited or non-existent in smaller cities and localities. Consequently, the intervention was described as having potential to fill a large treatment gap for PWD and caregivers. Despite this treatment gap, dementia care was considered to have a low priority in Swedish healthcare, wider society, and the political landscape, with PWD and caregivers described as being ‘left alone’ after receiving a dementia diagnosis:

*The problem is that there is no next step, it is somewhat different in different regions, what is available at different hospitals. But in general, there is no post-diagnosis plan, and that is a big problem.”* (Community stakeholder 1)

#### Specification (individual and communal)

HCPs and community stakeholders agreed that the overall intervention purpose is to support and help PWD and caregivers in everyday life to increase their well-being and quality of life:

*“To support the caregiver and PWD so they together can improve the life-quality of the PWD, I would say. So that the person* [with dementia] *can carry out activities in everyday life that he/she feels are manageable. You have a tool to be more active with activities that works, even though you have dementia.”* (Healthcare professional 1)

The intervention was viewed as a tool to communicate with the PWD – caregiver dyad in a pedagogic way, facilitate motivation, initiate activity, and provide structure. HCPs generally understood the intervention purpose, however some community stakeholders experienced difficulties understanding how they could endorse, refer, or guide the intervention.

#### Internalization

The intervention was perceived as having potential to positively impact both PWD and caregivers. For example, decrease caregiver burden, decrease/prevent mental health difficulties, improve quality of life among PWD and caregivers, maintain self-esteem, promote function among, and sustain continued community living among PWD. Wider potential impacts were also identified, including increasing dementia knowledge, normalizing dementia in society, and reducing long-term health and social care burden. The intervention was perceived to be a concrete tool for PWD, caregivers and HCPs:

*“I think the material provides a very concrete tool for both the caregiver and PWD to work with, and that is great. This is often what I see among caregivers, that they do not know how they can support* [the PWD] *or what they can do”.* (Healthcare professional 3)

Additional described benefits included the intervention serving to facilitate conversations about difficult situations and emotions between PWD and caregivers, such as dementia and mental health difficulties.

Whilst clear benefits were identified, potential negative impacts were described, mainly related to increased caregiver burden seen as a potential harm, given the potential negative impact of the intervention adding additional caregiving responsibilities:

*“In worst case, it* [the intervention] *will put pressure on caregivers who feel it is their responsibility to make sure that the PWD does all these things. And if you are caring for a person* [with dementia] *who experiences difficulties getting things started or has apathy, then the risk is that it* [the intervention] *turns into demands, pressure, and failure for the caregiver.”* (Healthcare professional 10)

Concerns were raised that the intervention will not be appropriate for all PWD, suggesting it being more suitable for people with mild-to-moderate dementia, rather than severe.

### Cognitive participation

Data supporting the NPT sub-constructs *Initiation*, *Enrolment*, and *Legitimation* were identified. No data was supporting the sub-construct *Activation*. Analysis resulted in two inductive categories: *Relational Interaction,* and *Definition and Evaluation of the Situation* (Fig. 2).

#### Initiation

Engaging and involving stakeholders responsible for decision-making such as managers was perceived as essential to drive the intervention forward and increase the likelihood successful implementation:

*“If I am thinking from my perspective, it must be a manager who makes sure that it* [the intervention] *happens.”* (Community stakeholder 7)

One community stakeholder suggested intervention endorsement could be done by local community stakeholders:

*“Involvement of local associations is needed, that the people responsible for local associations can go through this* [the intervention and its material] *and find out what they can do* [to help support the intervention].*”* (Community stakeholder 3)

#### Enrolment

A need to prepare both the dyad and HCP via the provision of a thorough intervention introduction was expressed. A need was also voiced for intervention information to be consistent, easy to understand, delivered at a slow pace, and repeated, or rephrased where necessary, especially for PWD. Explanations of potential intervention impacts and benefits were also perceived as important to increase dyad and HCP interest and motivation:

*“You must get an explanation why we are doing this* [the intervention], *what is important about it, and that we are helping their whole life together.”* (Healthcare professional 11)

However, lack of motivation and inability to take initiatives among PWD was mentioned as a barrier to intervention success:

*“The big problem is lack of ability to take initiative, many* [PWD] *have lost their ability to take initiatives completely, not only the motivation but the initiative itself. But it is incredibly different for different people, this* [the intervention] *is probably great for some people and quite bad for others.”* (Healthcare professional 8)

Intervention material text was considered too extensive and potentially overwhelming for PWD and caregivers, posing a barrier to using and sustaining the intervention.

#### Legitimation

HCPs and community stakeholders perceived themselves referring and endorsing the intervention via activities such as providing information to suitable PWD and caregivers. However, the only workforce that could perceive themselves as guiding the intervention were caregiver consultants:

*“I think that we, as caregiver consultants, could help to a certain extent and support caregivers in that* [guiding the intervention], *but they probably also need quite simple and clear information on what can be gained. Since there will still be a certain burden on the caregiver, they must see a profit from it. But I think as a caregiver consultant, I would be able to do that, be able to support the caregivers.”* (Healthcare professional 9)

#### Relational interaction

Establishing and maintaining a relationship between the dyad and intervention guide (the triad) was considered essential to facilitate confidence and commitment in the intervention and sustain its use. Face-to-face meetings, rather than online/telephone, were considered a facilitator for establishing a relationship and trust between the dyad and intervention guide, and important to help PWD understand the intervention and feel safe. Good communication within the triad was also raised as an important facilitator. Given it can be challenging and energy-demanding for PWD to establish several new relationships a preference was expressed for support to be provided by the same intervention guide throughout the intervention:

*“You establish trust and then you can take the rest* [of the intervention]. *It can be difficult with new contacts for many* [PWD] *or you can be suspicious and wonder about things. No, I think a person with whom you feel safe and with whom you can communicate, then I think it will go very well.”* (Healthcare professional 2)

#### Definition and evaluation of the situation

The importance of PWD recognizing their dementia was highlighted as a facilitator for taking part and engaging in the intervention, with those not recognizing unlikely to perceive an intervention need. Recognition and acceptance of their dementia diagnosis was seen as a pre-requisite for successful intervention engagement:

*“Of course, the condition* [to engaging in the intervention] *is that they admit that they have this* [dementia] *disorder. Because when they do not acknowledge this, they will not want to join* [the intervention] *…, then it is hard to get them to participate.”* (Community stakeholder 6)

A potential threat of conflict was also perceived between the PWD and caregiver in cases whereby the PWD does not want to participate in the intervention, whilst the caregiver would. HCPs did not suggest ways to manage the conflict, while community stakeholders held divergent views. For example, to use the intervention despite the PWD being unwilling, or alternatively re-allocate intervention resources to dyads whereby both members are willing to participate. Difficulties using the intervention were also recognized to arise if caregivers do not want to participate themselves, lack energy, or are unwell.

Stigma was also considered a barrier to intervention engagement. Both dementia and mental health difficulties were considered to be stigmatized in society, with stigma perceived as more prevalent among older PWD than younger peers. Older persons were considered as of a generation wishing to “fend for themselves”, preferring not to receive help from healthcare:

*“I think that younger people will find it easier to seek help compared to the elderly, because there is also some kind of stigma there. It is like dementia, it is a bit embarrassing, maybe you have not even told your children that the husband or wife has started to get disoriented and confused, and they are also the ones who want to fend for themselves. I think that those who are younger, the 60-plus people, they are probably seeking more help.”* (Healthcare professional 6)

However, the intervention was described as being important to raise awareness and normalize dementia and mental health difficulties:

*“It is so important that we dare to talk about mental illnesses and that we get there when it comes to dementia too.”* (Community stakeholder 2)

### Collective action

Data supporting the NPT sub-constructs *Interactional Workability*, *Relational Integration*, *Skill-set Workability*, and *Contextual Integration *were identified. Analysis resulted in one inductive category: *Prerequisites for Intervention Interaction* (Fig. 3).

#### Interactional workability

The intervention was described as a concrete way of working that could facilitate work practices being more organized, structured, and uniform among professional groups. Both HCPs and community stakeholders expressed that endorsing and referring people to the intervention could easily be integrated into their work practices:

*“We are an organization* [non-governmental organization working with dementia and caregivers] *that works a lot for caregivers … We could offer or be able to inform what is available, what support is available and so forth.” (Community stakeholder 1)*

Conversely, HCPs who perceived themselves guiding the intervention (i.e., caregiver consultants) expressed a need for significant work practice changes to operationalize the intervention into their everyday work:

*“I think it* [the intervention] *would take up a lot of my time. I think I would have to change my ways of working completely.”* (Healthcare professional 11)

#### Relational integration

Some HCPs (occupational therapists and dementia care consultants) were described as suitable intervention guides due to their competence, educational background, and dementia knowledge:

*“I think occupational therapists are great at breaking down activities ... That is what is needed, to really be able to break down activities to simpler difficulty levels, and to set new goals to achieve something*.” (Healthcare professional 7)

On the other hand, some health and social care professionals such as general practitioners, nurses, assistant nurses, and social welfare case managers were considered unsuitable to provide guidance in the intervention given lack of knowledge or education in dementia, which may lead to lack of confidence and trust. Lack of confidence was also held in some PWD and caregivers being able to manage participating, considering the intervention too complex:

*“My first spontaneous reaction was that the ones I know* [PWD and caregivers] *would probably not manage this*.” (Healthcare professional 6)

#### Skill-set workability

Knowledge and experience in dementia were described essential for intervention guides to operationalise the intervention, alongside empathy and genuine interest in the population:

*“It must be someone who have knowledge of dementia and provide support if problems arise, and at the same time is able to support the caregivers… Have common sense, being empathetic and have knowledge in dementia.”* (Healthcare professional 2)

A need was also expressed for rigorous intervention education and training for intervention guides. Furthermore, the importance of continuous supervision was highlighted.

#### Contextual integration

A need for additional resources in terms of funding, staffing, and time were described as essential to make the intervention work in practice. Some HCPs expressed being highly time-pressured, with the additional work of implementing a new intervention posing a barrier. Conversely, other HCPs and community stakeholders perceived the intervention as not very time-consuming or costly and potentially benefitting other healthcare areas, such as fewer people needing support for stress or sleep problems. The importance of involving and engaging politicians, policy-makers, and managers in the implementation of the intervention in practice was highlighted:

*“The thing is, if this* [intervention] *were to be presented to politicians and they would say ‘yes, this is what we are doing’, then we would be doing it. Then funding and resources would be invested so that this* [intervention] *would become visible and implemented .... Then you need someone who keeps the project going, who has a perspective in it, I think. Not just a small project that lasts six months or a year, but it requires long foresight. I also think that the same commitment from the region is needed, in my opinion, in order for us to do well*.” (Healthcare professional 18)

However, dementia care was considered having a low priority in Swedish politics and a need for politicians to start prioritizing dementia care and to stop neglecting PWD was expressed:

*“Above all, we have to get the politicians started so that they understand that we have to do something, because the costs today, so they are insurmountable. We are talking about 63 billion SEK back in 2012. I think you can add at least 10 billion to today's date for what these disorders cost just us in Sweden.”* (Community stakeholder 3)

#### Prerequisites for intervention interaction

A number of barriers and facilitators were identified that could affect enacting the intervention in everyday practice. Intervention guides were described as essential for the intervention to work, especially to guide caregivers to support PWD and provide support for caregivers’ own well-being:

*“When I meet caregivers, they are really crying out for help and support, sometimes they are just fumbling in the dark. Once I met a caregiver and I just listened to her, I did not say anything, because I did not know much then. But then she came back and she said ‘Thank you, I remember you. Everything was such chaos, but you were there and listened to me’. Maybe that is what it is all about, that caregivers get... you get to talk to a supervisor and get to feel this support.”* (Healthcare professional 16)

A need for PWD and caregivers to receive regular guidance throughout the intervention was expressed, with lack of motivation perceived as a potential barrier, especially for PWD. Different motivation levels and views on the intervention and its processes were perceived as potentially leading to conflicts between PWD and caregiver. Stakeholders also considered the complexity of the workbook material to be potential barrier to working with the intervention, leading to increased treatment burden:

*“It is a bit too much… When you have read through it* [the material] *… There is a lot to keep track of*.*”* (Healthcare professional 16)

## Discussion

Using NPT, we were able to understand how HCPs and community stakeholders perceived the intervention in comparison to current practice (*Coherence*), what relational work might be needed to individually and collectively build and sustain the use of the intervention in routine practice (*Cognitive Participation*), and how stakeholders needed to work to put the intervention into routine practice (*Collective Action*).

Findings suggest there was *Coherence* among HCPs and community stakeholders, with a treatment gap clearly identified alongside a need for psychological interventions for PWD and depression in Sweden. However, this perceived treatment gap is based on the perspectives of participants included in the present study. Given regional differences in dementia care in Sweden [58], this finding may not be transferable to regions not included in this study. The intervention was generally perceived as adding something new to current practice and importantly psychological interventions for depression are not mentioned in Swedish national dementia care guidelines [59]. Whilst a psychological treatment gap was acknowledged, there were disconfirming cases with some struggling to differentiate the intervention from current practice. Whilst some intervention elements (e.g., encouraging PWD to be more active) may be conducted in practice currently, a LI-BA intervention for PWD, guided by trained HCPs, has not yet been implemented in Sweden [35], and there is currently no workforce specifically trained to provide guidance to LI-CBT interventions [60]. To facilitate implementation, findings indicate a need to educate stakeholders to be able to differentiate the intervention from current practices [61] given lack of *Differentiation* could pose a barrier for intervention uptake. Stakeholders being unable to differentiate between their usual ways of working and new interventions is not uncommon [34, 62, 63]. Implementation strategies, such as active learning, group discussions [64], and hands-on training [65] may facilitate understanding and how the intervention differs from current practice.

An important barrier for potential intervention users related to *Cognitive Participation* was the presence of dementia-related stigma. Stigma in this population is highly prevalent and suggested to be related to discrimination in health services including lack of time for patients and shortcomings in training, which are associated with delayed help-seeking behaviours and poor quality of life in PWD [66]. Despite stigma representing a barrier to implementation, the potential of the intervention to raise awareness and normalize dementia was also expressed. In accordance with previous research, raising awareness, and providing education [67] and training in dementia are important steps for reducing stigma [64]. Stigma related to depression was also identified as a barrier, with wider evidence suggesting some older adults consider accessing depression treatment as a weakness [68]. As a potential result of felt and/or enacted stigma [69], an additional potential barrier for intervention uptake was dementia denial, with recognition and acceptance of dementia being perceived as essential for intervention engagement. Dementia denial has been shown to be an important barrier for seeking healthcare [70], making it more difficult for PWD and caregivers to initially understand the dementia diagnosis [71]. This intervention is developed for people with mild-to-moderate dementia, and denial is often experienced in the early dementia stage, therefore dementia denial may present a challenge for intervention uptake. For future implementation, it will be important to educate those endorsing and guiding the intervention in how to support PWD who are in denial of their diagnosis.

Whilst HCPs and community stakeholders perceived themselves endorsing and providing information about the intervention to suitable PWD, barriers to *Collective Action* were expressed. Identification of an appropriate HCP workforce to provide guidance to PWD and caregivers was considered challenging. In other high-income settings, there have been paradigms shifts in mental health service delivery. For example, the Improving Access to Psychological Therapies (IAPT) programme (newly renamed as NHS Talking Therapies for anxiety and depression) has been successfully implemented in England [72]. Within this service, a workforce of Psychological Wellbeing Practitioners has been trained specifically to guide LI-CBT within a stepped care service delivery model [73]. Similar initiatives have been developed in Canada [74], Australia [75, 76], and Spain [77]. Although an entire paradigm shift may not be feasible, task-shifting may represent an alternative solution [78] given knowledge and experience of dementia were considered essential skill sets. Occupational therapists, who are trained in supporting older adults to live independently in their homes, including assessing everyday activities, and supporting self-management interventions, may represent a suitable HCP group [79]. However, lack of time among all HCPs was reported as posing a barrier for providing intervention guidance, a common barrier to the implementation of task-shifting solutions [80].

Also related to *Collective Action*, some concern was raised about PWD and caregiver capacity to participate in the intervention due to factors such as burden, sickness, and lack of time and energy. Such concerns may indicate potential ‘gate-keeping’ [81]. Gate-keeping is not uncommon, with previous research showing reluctance among HCPs to refer older people with depression to mental health services arising from a belief that depression as an inevitable consequence of ageing [82]. Professional concern about caregiver burden has also been reported as a barrier for recruiting caregivers into research [83, 84]. Despite HCP’s intentions to minimize treatment burden, gate-keeping ultimately prevents people accessing appropriate care. Implementation strategies to increase stakeholders’ knowledge and awareness of evidence-based psychological interventions [7] including education and training [64] may be required.

### Strengths and limitations

To the best of our knowledge, this is the first study with the overall aim of adapting a guided LI-BA intervention for PWD and depression and their caregivers to enhance implementation potential for the Swedish context.

Following the MRC framework [22], and informed by NPT [31], we have successfully identified barriers and facilitators to intervention uptake which will be used to maximize future intervention implementation potential. Whilst NPT is a structured and theoretical informed approach, coding data in accordance with NPT sub-constructs was not always straightforward due to conceptual overlap. Also, adopting a deductive coding approach may have limited our understanding of barriers and facilitators not captured by the framework [54]. However, given the intervention is a self-help intervention built on dyadic (PWD-caregiver) and triadic (PWD-caregiver-HCP) interactions, we adopted a two-step analysis approach using the NPT coding manual [52]. Importantly, this two-step analysis approach allowed for inductive categories related to implementation potential that consider the work intervention users (i.e., PWD and caregivers) need to do to implement the intervention in practice. Our approach can be viewed as a logical development and expansion of NPT, which was originally developed for providing a framework for understanding HCPs perspectives on implementation of complex heath interventions in healthcare settings [85]. Given the self-help nature of the intervention, i.e., caregiver involvement in supporting the intervention and the intervention being situated within a triad [86], it is essential to consider barriers and facilitators to implementation related to all actors expected to take on an active role in the implementation. Consideration of how to design interventions to maximize implementation potential from both patients and HCPs is of particular importance when developing self-help/self-management interventions [87].

HCPs were recruited from five (of 21) regions across Sweden. Given wide variation in dementia care delivery between regions [88], this may limit transferability of results. Further, consideration of future hypothetical implementation of an intervention, rather than real-life implementation, may be difficult. However, this study was intended to be exploratory and potential implementers will continue to be engaged throughout intervention development, feasibility testing, and evaluation. Given many different HCPs and community stakeholders may be involved in future implementation (i.e., endorsing, referring, and guiding the intervention), we actively sought to involve stakeholders working in a variety of roles. This resulted in a variety, and sometimes conflicting and divergent, perspectives and suggestions on how the intervention should be developed. However, this is also a strength, as data can be applicable to a variety of different healthcare and community settings involved in future implementation. Multiple and sometimes conflicting perspectives have been reported in similar studies [34].

### Implications and further research

Early intervention development is rarely reported [89], making it unclear if or how implementation potential is considered, which may lead to future implementation failure [26]. Careful consideration of potential implementation barriers and facilitators throughout intervention development can help design context-specific interventions [26, 90, 91] Furthermore, it may help inform the development of appropriate implementation strategies [92], thereby reducing the risk of future implementation failure and research waste. Following MRC framework for complex interventions [22] and using NPT [31] during intervention development allowed us to explore how different stakeholders make sense of the intervention and integration into routine practice. This includes the identification of potential implementation barriers and facilitators that can be used to inform the identification of evidence-based implementation strategies to facilitate future implementation [93]. The importance of intervention users in implementation was recognised by HCPs and community stakeholders. Future research should explore barriers and facilitators to implementation from the perspective of intervention users themselves. We will continue to assess and manage potential implementation barriers through further development, feasibility and evaluation. However, it will also be important further understand important contextual factors, for example by adopting the Consolidated Framework for Implementation Research [94]. Our study was exploratory in its nature, involving different stakeholders. Future research may work more in-depth with those directly involved in intervention implementation, such as policy-makers and those guiding the intervention.

## Conclusions

Together with HCPs and community stakeholders and informed by NPT, we identified a number of barriers and facilitators for implementing a LI-BA intervention for PWD and depression in the Swedish context. The use of an implementation theory in the early intervention development phase will hopefully increase future implementation potential and potentially optimize effectiveness. Although this study focuses on barriers and facilitators to implementation from the perspective of key HCPs and community stakeholders, findings clearly illustrate the importance of considering the perspectives of all actors expected to take on an active role in the implementation. This includes the work PWD and caregivers need to do to implement the intervention in practice. Our approach may be useful in other studies on the implementation of complex interventions in which are expected to take an active role. This is of particular importance given the increasing focus on self-care health interventions worldwide [95].

### Supplementary Information

**Additional file 1.** Standards for Reporting Qualitative Research (SRQR)*.

**Additional file 2.** Focus Group Guide informed by the Normalization Process Theory.

## Data Availability

The datasets generated and/or analysed during the current study are not publicly available due to privacy or ethical restrictions but are available from the corresponding author on reasonable request.

## Abbreviations

BA: Behavioral activation
CBT: Cognitive behavioral therapy
HCP: Healthcare professional
LI-BA: Low-intensity behavioral activation
MRC: Medical Research Council
NPT: Normalization process theory
PAG: Public advisory group
PWD: People with dementia
