# Correction to: Healthcare and community stakeholders’ perceptions of barriers and facilitators to implementing a behavioral activation intervention for people with dementia and depression: a qualitative study using Normalization Process Theory

**DOI:** 10.1186/s12877-024-04829-1

**Published:** 2024-03-05

**Authors:** Frida Svedin, Oscar Blomberg, Anders Brantnell, Paul Farrand, Anna Cristina Åberg, Joanne Woodford

**Affiliations:** 1 Healthcare Sciences and E-Health, Department of Women’s and Children’s Health, Uppsala University, 751 85 Uppsala, Sweden; 2 Industrial Engineering and Management, Department of Civil and Industrial Engineering, Uppsala University, 751 21 Uppsala, Sweden; 3 Clinical Education, Development and Research (CEDAR); Psychology, University of Exeter, Perry Road, EX4 4QG Devon, UK; 4 Clinical Geriatrics, Department of Public Health and Caring Sciences, Uppsala University, 751 85 Uppsala, Sweden; 5 Medical Sciences, School of Health and Welfare, Dalarna University, 791 88 Falun, Sweden

After publication of this article, the authors reported that in the abstract, in the first sentence of the paragraph ‘Results’, ’Twelve’ should have read ‘Ten’

The original article has been corrected.

