# Supplementary material for: Healthcare and community stakeholders’ perceptions of barriers and facilitators to implementing a behavioral activation intervention for people with dementia and depression: a qualitative study using Normalization Process Theory
Source: BMC Geriatr. 2023 Dec 7;23:814. doi: 10.1186/s12877-023-04522-9 (PMC10702110; doi:10.1186/s12877-023-04522-9)
Supplement: Supplementary file 2 — Additional file 2. Focus Group Guide informed by the Normalization Process Theory. [file 12877_2023_4522_MOESM2_ESM.pdf]

Focus Group Guide informed by the Normalization Process Theory

1. What are your first impressions of the intervention?

2. How would you describe the purpose of the intervention?

3. How does the support provided in the intervention differ from the support people with dementia are currently receiving?

4. What impact do you think the intervention can have on people with dementia?

5. What impact do you think the intervention can have on informal caregivers?

6. What type of support do you think people with dementia need to understand what the intervention is and how it should be used?
   a) What difficulties might people with dementia experience when using the intervention?

7. What type of guidance do you think informal caregivers need to understand what the intervention is and how it should be used?
   a) What difficulties might informal caregivers experience when using the intervention?

8. What type of support do you think people in your organization need to understand what the intervention is and how it should be used?

9. Currently, there are no trained healthcare group who can provide the guidance in the intervention to the informal caregivers and people with dementia. Who or which group do you think is best at providing the guidance?
   a) How would the intervention affect the way of working for the workforce providing the support to people with dementia and informal caregivers?
   b) What can be done to ensure that the intervention becomes part of the routine practice for the workforce providing the support to people with dementia and informal caregivers?
   c) What resources would be needed to ensure that the intervention becomes a part of the routine practice for the workforce providing the support to people with dementia and informal caregivers?

10. How would the intervention affect your ways of working?
